# Do Peers Matter? Unhealthy Food and Beverages Preferences among Children in a Selected Rural Province in China

**DOI:** 10.3390/foods12071482

**Published:** 2023-03-31

**Authors:** Mi Zhou, Biyu Bian, Li Huang

**Affiliations:** College of Economics and Management, Shenyang Agricultural University, Shenyang 110866, China

**Keywords:** peer relationship, rural children, food preferences, fast food, snack foods, sugary drinks

## Abstract

With the growing problem of childhood obesity, unhealthy dietary preferences among children have become an issue of worldwide concern. This article examines the class-peer effect of these preferences using random class assignment data from a field survey. The results show significant positive peer effects for both unhealthy food and beverages preference. These results remained robust after controlling for endogeneity issues using instrumental variables. Further analysis of the mechanism of peer effect shows that the better the relationship between classmates, the greater the influence peers have on unhealthy diet preferences among rural children. The same peer effect is found in situations where it is easy for children to obtain unhealthy food and beverages. The analysis of heterogeneity finds that the peer effects of unhealthy dietary preferences are stronger for girls, older students, and obese students. This paper also discusses the role of parents and schools in mitigating the peer effect. This paper proposes policy recommendations for rural areas in China to improve dietary preferences in children. These results may also provide useful guidelines for policy in other developing countries.

## 1. Introduction

Food preferences, particularly in children, play a pivotal role in predicting human food choices [1]. Existing studies have shown that only a few taste preferences are innate, and most food preferences are learned through experience with food in social environments during childhood [2], meaning that food preferences can be modified and changed [3]. This study focuses on children and their unhealthy food and beverages preferences, as more than 340 million children age 5–19 years were overweight or obese in 2016 [4]. According to the Report on the Status of Nutrition and Chronic Diseases of Chinese Residents (2020), 18.9% of Chinese children and adolescents often drink sugary beverages. In 2020, 6.8% of children under 6 years of age in China were overweight, while 3.6% were obese, while 11.1% of children between 6 and 7 years of age were overweight and 7.9% were obese. Overall, the WHO estimates that 10.4% of Chinese children are obese as of 2022. Previous studies have shown that two to seven times more children eat unhealthy foods such as biscuits and sweets than eat fruits, vegetables and beans [5]. Unhealthy foods and beverages are particularly harmful to children and adolescents, as they may increase their risk of obesity later in life [6,7,8]. Healthy food preferences established in childhood and adolescence tend to continue into adulthood [9,10], which is extremely important not only for decreasing risks of noncommunicable diseases (NCDs, such as obesity, breast cancer, rectal colon cancer, ischemic heart diseases and stomach cancer) but also for decreasing risks of chronic diseases later in life [11,12,13,14].

Previous studies have emphasized factors influencing child dietary preferences, including sociodemographic status [15,16], emotions [17,18,19], personality factors [20], and environmental determinants, such as the advertising, marketing, and availability or accessibility of food [21,22,23,24,25]. In contrast, little is known about how peers influence food preferences.

Susceptibility to the peer effect increases throughout childhood and peaks during adolescence [26]. The past several decades of research reveal that peer interactions in childhood have both short-term and long-lasting effects on development [27,28]. Adolescents at this period of their lives want to seek peer approval and develop a social identity. Hence, peer influence is an important determinant in food selection [29]. In a snack choice experiment, children observing a peer eating an unhealthy snack were more likely to choose an unhealthy food as well [30]. Previous research has examined associations between adolescent healthy eating behaviors and suggested that adolescent friends exhibit similarities in dietary patterns [31]. Another study of 1220 adolescents demonstrated the direct impact of peer influence on saturated fat intake [32]. However, most of the relevant literature on the influence of peer effects on diet is descriptive statistical analysis or correlation analysis, and there are few studies that strictly identify the causal influence of peer effects and almost no research on the effect on rural children in developing countries.

The main objective of this study is to accurately identify the influence of peers on unhealthy dietary preferences in rural children. Firstly, this paper analyzes the correlation between the peer effect and unhealthy food preference in rural children, and it then carries out a robustness test after solving the reflexive problem using a random shift test and the instrumental variable method. Secondly, it considers the moderating effect of peer quality and food availability on the influence of the peer effect on food preferences. Finally, we further investigate the heterogeneity of peer effects of unhealthy dietary preferences of rural children in terms of gender, grade and obesity status. Some scholars pointed out that the reflection problem appears when identifying peer effects and then split the reflection problem into endogenous effects, exogenous (contextual) effects and correlated effects [33]. For endogenous effects, with other things being equal, the dietary preferences of individual students will change according to the dietary preferences of other students in the class. For example, students may adjust their dietary preferences as a result of eating with classmates [34,35]. Additionally, observation of and conversation with classmates may also affect individual students’ dietary preferences through shared cognition [36]. Exogenous (contextual) effects refer to how student dietary preferences may be affected by common environments (e.g., geographical environments, institutional environments). Correlated effects describe the tendency of children to exhibit similar characteristics because they share similar but unobservable personal characteristics. According to Manski [33], only endogenous effects generate a “social multiplier.” For example, when an intervention affects a group of students, this group of students will influence other students through peer effects, and other students will in turn affect this group of students.

In this case, the exogenous and correlated effects must be excluded to accurately identify endogenous effects. To exclude possible exogenous effects, as much information as possible (including the characteristics of schools, classes and teachers) should be added into the econometric model as control variables [37]. In addition, the instrumental variable method can be used as a solution for possible omitted variable bias. As for the possible correlated effects, eliminating student choice behavior is the most intuitive method. Fortunately, the children in the sample used for this study are from schools in which students are randomly assigned to classrooms, which reduces the problem of sample self-selection.

To the authors’ best knowledge, no study has analyzed the peer effects of unhealthy dietary preferences among children in developing countries. After excluding exogenous and correlated effects, this study found that peer preferences for unhealthy food and beverages significantly increase rural children’s preference, and this result remains robust after a series of robustness tests.

This study also contributes to the understanding of peer effects of children’s unhealthy dietary preferences by exploring potential mechanisms. It is found that high-quality relationships can significantly increase the effect of peer effects on unhealthy dietary preferences among rural children. The availability of unhealthy food and beverages (i.e., the ease of purchase) also plays an important role.

Finally, this study also analyzes differences in the peer effects by gender, grade and obesity status. In addition, the roles that parents and schools can play are also considered in this paper. This study supplements existing literature and provides a means of understanding the food preferences of Chinese children. Unlike previous studies, this paper obtains random class assignment data and uses scientific statistical methods.

## 2. Literature Review, Conceptual Framework and Hypotheses

### 2.1. Literature Review

Previous studies have shown that peers play an important role in the dietary behavior of rural children, and peer effects have an impact on unhealthy food preferences of rural children [38]. A randomized controlled experiment showed that after seeing peers choose unhealthy food, children became more likely to choose unhealthy food, resulting in negative peer behavior [30,39]. However, some studies have found that the healthy diet encouragement of their peers will reduce the intake of unhealthy foods such as snacks and beverages, thus encouraging children to make healthier dietary choices [40].

Many studies have proved the influence of peer relationships [41] and food availability [42] on unhealthy food preferences in rural children. In addition, the influence of the peer effect on unhealthy food preferences differs according to children’s individual characteristics, parents’ understanding of nutrition, and the style of nutrition education in schools. Many studies have focused on the impact of gender, age, obesity, peer sensitivity, and other characteristics on the diets of rural children. Additionally, many studies have shown that there is a significant negative relation between gender and unhealthy diet, and that boys are more likely to eat unhealthy food than girls [29]. In terms of age, children in middle school are more likely to be affected by their peers’ unhealthy food preferences [40,42]. In an experiment, it was found that the presence of peers affects the caloric intake and healthy food choices of overweight children [43]. Another study showed that children who were sensitive to peer influence consumed more snacks than children who were not [44]. At the same time, preference for unhealthy food is also affected by factors such as parental education level and nutrition knowledge. The more nutrition knowledge mothers have, the more likely children are to prefer healthy food [45,46]. Among the ways in which schools carry out nutrition education, video clips and brochures play an important role in increasing dietary nutrition knowledge and improving unhealthy eating behaviors [47].

In recent years, many scholars have focused on the relationship between the peer effect and eating behavior of rural children, but few have studied and analyzed the mechanism by which the effect is exerted. Therefore, this paper not only analyzes the relation between peer effect and rural children’s unhealthy food preferences but also analyzes the causal relationship between them. Secondly, this study carried out a random class test and explored the mechanism of the peer effect on unhealthy food preferences of rural children. Thirdly, considering the particularity of rural children in China, this paper analyzes the heterogeneity of peer effects of the unhealthy food preferences of rural children in terms of the characteristics of children, parents, and schools, and provides targeted reference programs for improving rural children’s eating behavior.

### 2.2. Conceptual Framework and Hypotheses

Children are motivated to voluntarily follow the social norms of peer groups, so they are likely to adopt eating attitudes and preferences similar to their peers. This behavior is positively correlated with unhealthy eating preferences [48]. Research on unhealthy eating behavior of rural children has found that there was a relation between peers and rural children eating unhealthy food, especially snacks with high saturated fat content [49]. Some experiments have also confirmed that the intake of unhealthy snacks by rural children in the presence of their peers is higher than that of their mothers [50]. Therefore, this paper proposes the following hypothesis:

**Hypothesis 1 (H1).**  *Peer preference for unhealthy food determines unhealthy food preferences in rural children.*

Studies have shown that peers not only affect the food choices and intake of rural children but also play an important role in their unhealthy food preferences [41]. Some experimental studies have shown that when children know each other well, the social facilitation of eating occurs [51]. An individual’s total caloric intake is therefore related to that of their friends [44,52]. Therefore, this paper proposes a second hypothesis:

**Hypothesis 2 (H2).**  *Peer relationship moderates the causal relationship between peer effect and unhealthy food preference of rural children.*

Many studies have confirmed that food availability plays an important role in peer effects on unhealthy food preferences of rural children. The closer a school is to a convenience store, the greater the chance that children will buy unhealthy foods, such as snacks and sugary drinks with their peers during school [42]. An experiment also shows that if there are fast food restaurants near the school, teenagers are more likely to be influenced by their peers and have a preference for unhealthy food [53]. Therefore, this paper proposes a third hypothesis:

**Hypothesis 3 (H3).**  *Food availability moderates the causal relationship between the peer effect and unhealthy food preferences in children.*

**Figure 1:**
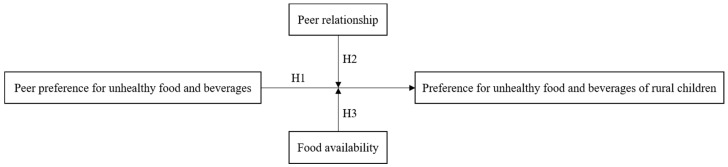
Conceptual Framework.

The conceptual framework of this study is shown in Figure 1.

## 3. Methodology

### 3.1. Data

These data are from a research project designed and implemented by the Agricultural Economic Theory and Policy Research Center of Shenyang Agricultural University. The study aims to understand the current situation of rural Chinese children in terms of dietary nutrition, physical and mental health, social adaptation, mobile phone and Internet use. The survey was carried out in Henan, China, which is a primary province for migrant workers in central and western China. Henan is a traditionally agricultural province and has the largest population in central China.

The survey method used in this paper is census survey. The survey took place at 8:00 AM on 1 December 2021, in Xinzhuang Town, Fan County, Puyang City, Henan Province, and involved primary and secondary school students in Xinzhuang Town. The enrollment rate of the school-age population and the coverage rate of nine-year compulsory education in Xinzhuang town are both 100%. As children in the younger grades have weak reading comprehension skills, they could not complete the questionnaire independently, so only children from grades 4 to 9 were investigated.

Throughout the investigation, the research group received strong support from the central school in Xinzhuang and from the teachers in charge of each school. A total of 55 classes in 15 primary schools and 1 middle school were investigated, and a total of 1817 rural children were investigated. A total of 1817 parents and 55 teachers were involved (parents or guardians of each student and all of the teachers in charge of the survey). The questionnaire structure includes three main parts: child questionnaire, parent questionnaire and head teacher questionnaire. The questionnaires include a rich set of questions about the children’s development, family background, and class and school environment. They were pretested before fieldwork. To improve response quality, children filled out the questionnaire with trained survey coordinators and teachers present to help them with any questions or difficulties they had.

Before the formal investigation, the project team contacted the head teacher and parents and asked the parents to sign an informed consent form. All children with parental consent were eligible to participate in this study. The project team also promised to keep the personal information of all children involved in the survey confidential. In addition, the investigators of the project team and the teachers involved in the survey established a one-to-one working group. If it is necessary to expand the content of the study, conduct questionnaire quality inspection or do a return visit, the researchers can contact the rural children interviewed in each class in a timely manner through the teachers using this group. For the question of who the investigator is and how the study was conducted, the researchers from the project team and the head teacher jointly served as the investigator for each class, and we conducted a questionnaire survey and quality control training for all the teachers participating in the survey. The questionnaire survey was conducted during the students’ study hall and did not take up their normal classroom time. After all the questionnaires were completed, the teacher in charge of the class submitted them to the questionnaire quality controller on the project team for review and feedback. The teacher in charge of the class then organized the students to revise the questionnaire to complete the survey.

### 3.2. Variables

#### 3.2.1. Preference for Unhealthy Food and Beverages

The dependent variable of this paper is preference for unhealthy food and beverages among children in a selected rural environment. Unhealthy food refers to food with high calories, sodium and sugar, and low protein, essential fatty acids, vitamins, minerals and fiber [18]. Snack foods, fast foods, soft drinks and sugary fruit drinks meet the above characteristics of unhealthy food. Although these foods have low nutritional content, their taste, packaging and advertising are appealing to children and adolescents [54]. Therefore, this paper selects these unhealthy food categories (snack foods, fast foods, soft drinks and sugary fruit drinks) for the study [55,56,57]. The paper mainly focuses on three categories: fast food (such as fried chicken, pizza, or hamburgers), snack foods (such as potato chips, pretzels, or French fries) and soft drinks and sugary fruit drinks [58]. Students were required to select “dislike very much,” “dislike,” “neutral,” “like,” or “like very much” according to their personal preferences. The current paper re-assigned the above options and generated values between 1 and 5 [59,60]. As shown in Table 1, the preference of rural children for these three types of unhealthy food is snack foods > fast foods > soft drinks and sugary fruit drinks.

The independent variable of interest in this study is the unhealthy food and beverages preferences of other students in the class, which is represented by the average unhealthy food and beverages preferences in the class (excluding the children interviewed): the average fast food preference degree, the average snack preference degree and the average beverages preference degree at the class level.

#### 3.2.2. Control Variables

Based on the existing research and the availability of data, this paper selects personal, family, class and school control variables. Student characteristics include: gender, age, whether or not the child is an only child, boarder, transfer student, LBC (left-behind child), and adjusted self-rated health. Family characteristics include: parent education, parent occupation, and family economic status. Class and school characteristics include: class size, head teacher career duration, gender, age, and whether the school provides a “nutritious lunch” or regularly educates students about nutrition. Studies have shown that adolescent decision making about food choices is also influenced by psychosocial factors such as emotions [17,18,19]. Therefore, this study controlled for students’ depression scores in the regression equation. Depression scores were measured by the CES-D scale. The CES-D scale includes 20 items and is scored on a Likert scale, with four possible answers corresponding to how often the respondents experienced a given emotion within the past week: “rarely or none of the time (less than 1 day)”, “some or a little of the time (1–2 days)”, “occasionally or a moderate amount of time (3–4 days)”, and “most or all of the time (5–7 days)”. Possible scores range from 0 to 60, and higher scores indicate poorer mental health. In addition, prolonged use of screen-media devices has been described as a significant contributor to poor eating patterns in children and adolescents [1,62], so a dummy variable was added to the equation to measure whether students play online games.

### 3.3. Econometric Models and Empirical Strategies

The following econometric model is used to estimate the peer effects of preferences for unhealthy food and beverages:

(1) $${Preference}_{i}=\beta_{0}+\beta_{1}\bar{{Preference}_{j}}+\beta_{2}X_{i}+D_{s}+u_{i}$$

where the dependent variable ${Preference}_{i}$ is the dietary preference of student *i*, representing the preference for fast food, snack foods and soft drinks and sugary fruit drinks, respectively; $X_{i}$ represents the control variables; $u_{i}$ is the error term; $\bar{{Preference}_{j}}$ represents the average dietary preference degree of other students in the same class, which is calculated by the following formula:

(2) $$\bar{{Preference}_{j}}=\frac{1}{N-1}\sum_{\begin{array}{c} j=1\\ j\neq i \end{array}}^{N}{Preference}_{j}$$

In particular, as the random class assignment is conducted within a school and students may nonrandomly select their school, school fixed effects $D_{s}$ are included in the regressions to control for all school-level factors in the cross-sectional data that may influence student school selection decisions. Since dietary preferences in this paper are ordinal variables, both Ordered Probit (Oprobit) and OLS regression were used in model (1). In this paper, the food preference of rural children is scored using the Likert scale. The higher the score from 1 to 5, the greater the preference for the food. The score is both an ordinal variable and a continuous variable. Therefore, it is suitable to use Oprobit and OLS regression, and the two regressions also verify the robustness of the results in this paper. Standard errors are clustered at the class level, accounting for association in outcomes for students in the same class.

## 4. Estimation Results

### 4.1. Preliminary Results

#### Peer Effect on Preference for Unhealthy Food and Beverages among Rural Children

In the basic regression, this paper adopts Oprobit and OLS models to estimate the parameters of the survey sample. Table 2 reports the estimation results of the Oprobit model and the OLS model. For all three unhealthy food categories, the coefficients of the peer effect were significantly greater than 0. This shows that whether based on the OLS model, which treats dietary preference as a continuous variable, or the Oprobit model, which addresses intrinsic ranking, peers played a significant positive impact on the unhealthy dietary preferences of rural children.

Specifically, from the regression results of Oprobit and OLS models, the higher the preference peers had for fast foods (FF), the greater the preference rural children would have for fast foods (FF). The regression coefficients of the two models are 0.564 (*p* < 0.01) and 0.532 (*p* < 0.01), respectively. Peer preference for snack foods (SF) significantly affects preference for snack foods (SF) among rural children. The regression coefficients of the two models are 0.353 (*p* < 0.05) and 0.296 (*p* < 0.1), respectively. Peer preference for soft drinks and sugary fruit drinks (SD/SFD) also has a significant positive impact on the preference for soft drinks and sugary fruit drinks among rural children. The regression coefficients of the two models are 0.294 (*p* < 0.01) and 0.290 (*p* < 0.01), respectively.

Additionally, school-based nutrition education had a significant impact on reducing the intake of all three types of unhealthy foods and drinks. The differences between different educational styles will be discussed further in Section 4.3.3.

### 4.2. Core Results

#### 4.2.1. Robustness Test

##### Random Class Assignment Testing

To verify the randomness of class assignment for this sample, a balancing test was conducted between the predetermined characteristics of the head teacher (duration of teaching career, professional title, gender and age of the head teacher) and the background characteristics of the children and their parents or family. The results are shown in Table 3. Each cell represents a separate regression that regresses each predetermined characteristic of the head teacher on characteristics of the children, parents, and families. The results show that most regression coefficients are not significant. This suggests that there is no significant relation between teacher characteristics and the characteristics of the children, parents, and families within the school sample.

**Figure 2:**
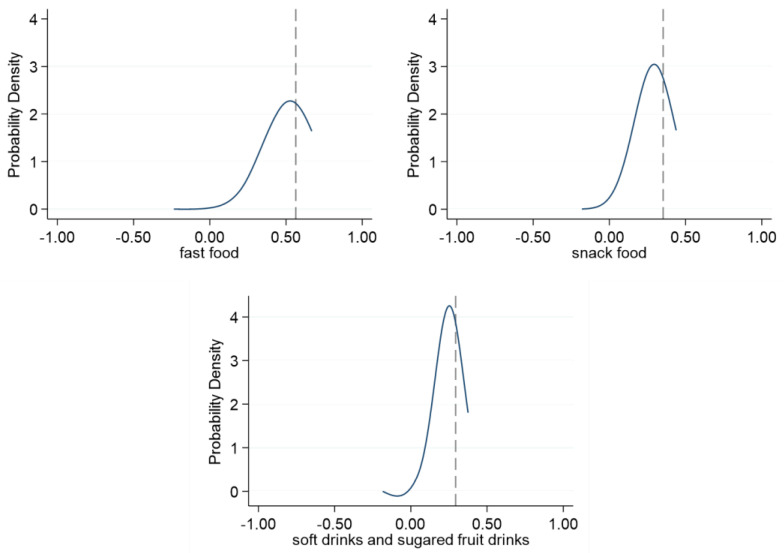
Density function diagrams of peer effect coefficients.

Another robustness test was performed to further verify that non-random class assignment did not occur within the sample. We randomly removed schools from the sample and observed whether there was a significant change in the regression results. If the students are in fact randomly assigned to classrooms, then the regression results using the random subsample should not deviate much from the benchmark regression results. To ensure the size of the sample, two schools were randomly deleted each time, and 500 regressions were performed. Figure 2 shows the density function of the peer effect coefficients in the random subsample regression. The black dotted line is the coefficient value of the benchmark regression. After random deletion, the coefficients of the samples were concentrated near the value of the benchmark regression coefficient, indicating that the benchmark regression results are not biased due to the inclusion of non-random data.

##### Instrumental Variable Regression

Instrumental variable estimation is also used to deal with the possible endogeneity problem and test the robustness of the benchmark regression. This study selects variables that only affect the dietary preferences of peers, rather than the individual in question, as instrumental variables, such as the class-averaged parental characteristic variable. That is, parents can only affect the peers of their children by affecting the behaviors or preferences of their own children. The instrumental variable used in this study is whether peer parents pay attention to the nutrition facts table when purchasing packaged foods (such as bread, milk, snacks, or drinks). When buying food, if parents pay attention to the nutritional ingredient list on the package, it shows that they place importance on the nutritional value of the food their children eat, impacting the dietary preferences of their children.

Next, instrumental variables are used to deal with endogeneity problems caused by unobservable factors under the framework of Extended Regression Models (ERMs). As the dependent variable in this paper is the sequence variable, the Eoprobit command is used for estimation. The two-stage least squares method (2SLS) is also used to carry out the robustness check. Table 4 lists the estimation results of the ERMs of the instrumental variables and the 2SLS method, respectively. The results show that the coefficients of the core explanatory variables were all significantly positive, indicating that peer preference for unhealthy food and beverages significantly improves the preference for such foods and beverages in the sample.

The coefficients of the instrumental variables in the first stage of the two-stage least squares method are all significantly negative, which further illustrates the association between the instrumental variables. The Kleibergen–Paap rK Wald F statistic for measuring weak instrumental variables is far greater than the critical value (16.38) at the 10% significance level, so the weak instrumental variable hypothesis can be rejected [63]. Additionally, the coefficients estimated by the instrumental variable method are larger in absolute value than the baseline estimates, which indicates that the endogeneity problem has led to underestimation of the peer effect. However, instrumental variable estimation does not change the conclusions obtained by the benchmark estimation, which show the robustness of the basic regression results in this paper. Hypothesis 1 has been verified.

#### 4.2.2. Potential Mechanism Analysis

This study finds positive and significant effects of peers on unhealthy dietary preferences among rural students. In this section, we explore potential mechanisms and, in particular, focus on peer relationships and food availability.

##### Peer Relationship

In this study, two questions from the student survey are used to assess the relevance of this mechanism: “I think I have a lot of friends (peer quantity),” and (ii) “I have a good relationship with my classmates (peer quality)” to measure peer relationships. Students are asked to rate to what extent they agree with the statement on a scale from 1 (strongly disagree) to 5 (strongly agree).

The first two parts of Table 5 report the estimation results. For both FF and SF, the interaction terms of “peer relationship” and peer unhealthy food preferences are significantly positive, indicating that when interactions between peers improve, peer appetites and preference for “delicious food” will affect the children more. Hypothesis 2 has been verified.

##### Food Availability

Stores near school are major sources of unhealthy food for students. Students often visit these stores in groups on their commute to school and during lunch breaks. This paper measures the availability of unhealthy foods through the question “Is there a grocery store within 500 m of the school? (1 = yes, 0 = no)” in the teacher questionnaire. Judging from the coefficients of the interaction terms in the last part of Table 5, having a grocery store near the school significantly increases peer influence on FF and SD/SFD, particularly the latter. The results of this paper show that the availability of unhealthy foods such as snack foods, soft drinks and sugary fruit drinks is very important when determining whether children will show healthy or unhealthy food preferences.

The mechanism of human-to-human transmission of dietary preferences relies on both information sharing and behavioral imitation. When children see their peers eating fast food or drinking carbonated or sweetened sodas, they may feel the urge to purchase the same food or drink. Therefore, convenient shopping conditions near the school increase opportunities for students to consume these unhealthy foods or beverages, creating an even greater peer effect. Hypothesis 3 has been verified.

### 4.3. Contrasting Hypotheses Results

#### 4.3.1. Individual Heterogeneity Analysis

The main findings of this study, which are reported in Table 2 and Table 4, capture the average peer effects on unhealthy food and beverages preferences. To further compare and investigate the heterogeneity of peer influence among rural children with different genders, grades and obesity levels, interaction terms of gender, grade (1 = junior high school, 0 = elementary school) and obesity variables were constructed. These interaction terms were also introduced for analysis based on the regression model of instrumental variables. The results are shown in Table 6.

The first part of Table 5 shows that the coefficients on the “peer preference × gender” interaction are statistically significant for both SF and SD/SFD. Compared with boys, peer effects have a greater impact on preferences for SF and SD/SFD in girls. However, there is no significant gender difference in peer effects for FF. The second part of Table 6 shows that only the peer effect of the SD/SFD preference is significantly different among students of different grades. Compared with primary school students, junior high school students have a preference for SD/SFD that is more likely to be influenced by their peers. Junior high school students were also more influenced by peers for their FF and SF preferences, but this effect was not significant.

According to the results in the third part of Table 6, the influence of the peer effect on FF is significantly different among students with different degrees of obesity. Compared with non-obese students, preference for FF in obese students is more easily influenced by peer preference for FF. For the other two categories of unhealthy foods, there is no significant difference between obese and non-obese children.

#### 4.3.2. Heterogeneity Analysis of Parents

This paper examines whether parental dietary knowledge (DK) moderates peer influence on unhealthy dietary preferences (estimation results reported in Table 7). A total of 17 questions related to DK are used to measure the DK of the parents of the children interviewed (Table A1 in Appendix A). Each question requires the respondent to select “strongly disagree,” “disagree,” “neutral,” “agree,” or “strongly agree” based on their personal circumstances. Based on the existing literature [64], the above options were re-assigned, generating a value between 0 and 2 that indicates the correctness of the answer (“T/F” column in Table A1): a score of 0 represents “strongly disagree” and “disagree”; 1 represents “neutral” and 2 represents “agree” and “strongly agree.” Finally, the total score of DK is obtained by summarizing the scores for the 17 questions. Among them, the highest score for DK is 34 with a high score indicating abundant DK. The average DK score of parents of children in the sample is 22.366. Xiang et al. used the same questionnaire to measure the DK of Chinese adults in 2021, and the average DK score was 26.855, which was higher than that of parents of the children in this sample.

According to the coefficient of the interaction term in Table 7, parental nutrition knowledge can significantly alleviate the influence of peers on rural children for SF, but the moderating effect of the other two unhealthy food and beverages categories is not significant. Parents play the role of health promoters, role models, and educators in the lives of their children, influencing their food cognitions and choices. Research has illustrated that a number of parental behaviors (for example, active guidance and education) are strong correlates of child food consumption behavior [65]. The guidance and education parents can provide depend on their knowledge. The results of this research further verify that parental dietary knowledge can indeed reduce the consumption of junk food by rural children.

#### 4.3.3. Heterogeneity Analysis of School

As mentioned in the above baseline regression analysis, school nutrition education has a significant negative relation with preferences for three types of unhealthy food and beverages. This section aims to compare the differences in the effects of different education methods. Sample schools educate children about nutrition in three main ways: classroom education, promotional videos or brochure education, and practical activity education. In this study, three 0–1 dummy variables were used to represent different methods and incorporate them into the econometric model.

The results in Table 8 show that the effects of classroom education on unhealthy diet preferences among rural children are all negative but not statistically significant. This may be because classroom education is the method adopted by most schools, and the difference between schools is not obvious. Promotional videos or brochure education has a significant negative relation with preference for SF and SD/SFD. Surprisingly, practical activity education has a significant positive correlation with preference for two types of unhealthy foods, FF and SF. When asked about the content of these activities, school administrators stated that these schools all participated in a food safety and nutritional health education practice activity for teenagers sponsored by a fast-food company. Although such activities allow young people to understand the source of ingredients, the process of food processing and cooking, and information about nutrition, they also indirectly promote fast food to children.

## 5. Discussion

### 5.1. Further Discussion

The results of this study show that unhealthy food and drink preferences have a significant positive peer effect, and the better the peer relationship, the greater the impact on unhealthy food and drink preferences of rural children.

The unhealthy food preferences of rural children are greatly affected by their peers’ preferences for snacks, fast food, and sugary beverages. Some studies also show that children are more likely imitate the behaviors of their close friends [30]. Additionally, studies show that eating habits and nutrition cognition are significantly affected by peers [66]. A randomized controlled experiment showed that displaying healthy food in the media does not lead children to choose healthier food, but when most of their peers did not like certain fruits, the probability of children not liking that food would increase [67]. The results of this study are consistent with those of previous studies.

This paper not only analyzes the influence of the peer effect on unhealthy food preferences but also further analyzes its potential mechanism. It has been found in the literature that the relationship between students and their friends may influence the size of the peer effect [68,69,70,71]. Food availability plays an important regulatory role in the interpersonal transmission mechanism of food preference. In response to the obesity epidemic, governments in some developed countries have regulated the sale of high-calorie, low-nutrient-density snacks and beverages in schools, for example by using taxes or bans [72]. At the same time, the availability of different foods was found to be associated with an increased consumption of available foods [73,74,75]. Therefore, it is important to control the source of unhealthy food. For example, some schools in the U.S. adhere to closed-campus policies that do not allow students to leave campus during lunchtime [76].

This study found that the peer effect of rural children’s unhealthy food preferences is heterogeneous due to their individual characteristics (such as gender, age and obesity). Firstly, gender had a significant positive impact on preference for fast foods (FF) and soft drinks and sugared fruit drinks (SD/SFD). Food preferences have already been linked to gender in the literature. Cooke and Wardle [77] found that boys liked fatty and sugary foods, meat and processed meat products more than girls did. In a study by Caine-Bish and Scheule [78], it was stated that boys showed a greater preference for meat, fish, and poultry than girls, while girls liked vegetables and fruits more than boys. Our results are consistent with the existing literature, given that fried chicken, burgers and other fast food are typically meat-based. Secondly, although it has been found in the literature that younger students are more susceptible to peer effects [66], this difference is more present in peer effects on cognitive abilities such as academic performance [79]. In terms of food consumption, older students are more likely to be influenced by peer effects because they may have more money available to them. Indeed, it has been confirmed by the literature that adolescents are more independent and also have more money to spend than their younger peers [80]. Other studies in the literature also point out that adolescents are more sensitive to the availability of junk food than younger children. This may be because adolescents are able to leave school campuses to access other sources of junk food more easily (i.e., in convenience stores or fast-food restaurants) [72]. Thirdly, existing research suggests that higher rates of fast-food consumption are connected to increasing rates of severe obesity, thus creating a vicious cycle [81], which is consistent with the research results of this paper.

### 5.2. Limitations and Future Research Directions

#### 5.2.1. Limitations

This study has several limitations. First, since this is a cross-sectional study, the long-term effects of the peer effect cannot be observed. Secondly, due to data limitations, there may be other confounding factors that have not been observed, such as personality characteristics and eating habits.

#### 5.2.2. Future Research Directions

It is generally believed that the behavior of peers or friends affects the behavior of individuals, which is important in analyzing the herd effect of rural children’s food consumption and group obesity. In future research, more specific and objective questionnaires and panel data are needed to eliminate the influence of unobservable factors and verify the long-term impact of peer effect on rural children. It is worth further exploring the impact of different aspects of peer effects such as peer relationship network and peer quality on rural children’s food preferences, food consumption, nutrition intake, and physical and mental health. This will provide theoretical and empirical reference for improving the health of rural children in developing countries.

### 5.3. Policy Implications

These findings make several contributions to the literature and have policy implications. First, understanding these mechanisms provides an opportunity to implement educational policies designed to reduce student consumption of unhealthy food. For example, convenient shopping conditions near schools can significantly enhance the peer effect of unhealthy dietary preferences. One implication may be that the sale of unhealthy food and beverages both from and around schools should be restricted. Second, this study calls on schools and families to pay attention to the problem of unhealthy food preferences of rural children. These groups play an important role in encouraging rural children to develop good eating habits, because rural children who show strong food preferences are more likely to develop inadequate or unhealthy diets. Therefore, when designing intervention programs aimed at improving the unhealthy food preferences of rural children, we must consider the dietary behavior of peers.

## 6. Conclusions

This paper sheds light on how rural children are influenced by peer preferences for unhealthy food and beverages. A random assignment of students was used to waive concerns about self-selection problems. Instrumental variables were further used to deal with endogeneity problems caused by unobservable factors under the framework of Extended Regression Models (ERMs).

These results show that peers in the classroom significantly increase rural children’s preference for unhealthy food and beverages. By exploring the potential mechanisms at play, this study finds evidence that with better peer contact, peer preference for unhealthy food and beverages will have a greater impact. Additionally, the availability of unhealthy food and beverages greatly increases the peer effect. In addition, there was heterogeneity in the influence of peers on rural children’s preference with different individual characteristics. Girls, older students and obese students were more influenced by their peers.

These findings provide useful information for school administrators who seek to optimize nutrition and health education. Promotional videos or brochure education and practical activity education are more effective than classroom education. Therefore, schools should focus on improving nutrition and health education and training methods for rural children, and the educational authorities should prohibit the provision of education by food producer entities.

Previous studies have shown that in rural China, caregivers generally have a low level of dietary knowledge (DK) [82]. Therefore, this paper also provides insight for policymakers regarding how to improve the DK of rural child caregivers. This study shows that the DK of rural caregivers can significantly mitigate the peer effect of unhealthy dietary preferences. In view of the fact that most of the caregivers of rural left-behind children are grandparents with lower levels of education, the government needs to take various effective measures to improve their awareness of the harm of unhealthy food such as junk food, snacks and sugary drinks on the health of rural children.

## Figures and Tables

**Table 1:** Summary Statistics.

Variables	Definition	Mean (1)	SD (2)	Obs. (3)
Dependent variables				
Preference for Fast Foods	1 = dislike very much; 2 = dislike; 3 = neutral; 4 = like; 5 = like very much	3.473	1.071	1817 ^4^
Preference for Snack Foods	1 = dislike very much; 2 = dislike; 3 = neutral; 4 = like; 5 = like very much	3.535	0.998	1817
Preference for Soft Drinks and Sugary Fruit Drinks	1 = dislike very much; 2 = dislike; 3 = neutral; 4 = like; 5 = like very much	3.261	1.106	1817
Control variables				
Gender	1 = male; 0 = female	0.495	0.500	1817
Age	continuous variable (year)	11.331	1.496	1817
Only Child	1 = yes; 0 = no	0.048	0.214	1817
Boarder ^1^	1 = yes; 0 = no	0.165	0.371	1817
Transfer Student ^2^	1 = yes; 0 = no	0.205	0.404	1817
LBC (Left-Behind Children) ^3^	1 = yes; 0 = no	0.379	0.485	1817
Adjusted Self-Rated Health	1 = very healthy/healthy; 0 = very unhealthy/unhealthy/neutral	0.649	0.477	1817
Depression Scores	CES-D Scores	15.810	9.267	1817
Online Game Player	1 = yes; 0 = no	0.845	0.362	1817
Parent Education	1 = junior high school or above; 0 = other	0.125	0.331	1817
Parent Occupation	1 = farmer; 0 = other	0.613	0.487	1817
Family Economic Status	annual family income (taking the logarithm)	10.764	0.535	1817
Class Size	Number	38.008	12.972	1817
Teacher Career Duration	continuous variable (taking the logarithm)	2.103	1.115	1817
Teacher Gender	1 = male; 0 = female	0.200	0.400	1817
Teacher Age	continuous variable (year)	35.656	8.387	1817
Nutritious Lunch	1 = yes; 0 = no	0.210	0.407	1817
Nutrition Education	1 = yes; 0 = no	0.909	0.288	1817

^1^ Boarder refers to students studying and living at boarding schools. ^2^ Transfer students are students who have not graduated but have transferred to a different school. ^3^ LBC (Left-behind children) refers to the children who stay in the countryside while one or both of their parents migrates from the countryside to the city to engage in non-farm work for 6 or more months [61]. ^4^ Of the 1817 rural children, the proportions of children in grades 4, 5 and 6 of primary school are 26.42%, 25.81% and 28.07%, respectively, and the proportions of children in grades 7, 8 and 9 of secondary school are 6.44%, 6.93% and 6.33%, respectively.

**Table 2:** Estimation results of the peer effect on preference for unhealthy food and beverages among rural children.

Variables	Oprobit			OLS		
	FF ^1^	SF ^2^	SD/SFD ^3^	FF	SF	SD/SFD
Peer preference for FF	0.564 ***			0.532 ***		
	(0.145)			(0.137)		
Peer preference for SF		0.353 **			0.296 *	
		(0.166)			(0.151)	
Peer preference for SD/SFD			0.294 *			0.290 *
			(0.156)			(0.156)
Control variables	Yes	Yes	Yes	Yes	Yes	Yes
School fixed effects	Yes	Yes	Yes	Yes	Yes	Yes
Observations	1817	1817	1817	1817	1817	1817
Pseudo R-squared/R-squared	0.032	0.020	0.029	0.083	0.051	0.081

^1^ FF refers to fast foods, ^2^ SF refers to snack foods, ^3^ SD/SFD refers to soft drinks and sugary fruit drinks. Standard errors are clustered at the class level and reported in parentheses. Significance: * *p* < 0.10, ** *p* < 0.05, *** *p* < 0.01.

**Table 3:** Balancing test.

Variables	Teacher Career Duration	Teacher Age	Teacher Professional Title	Teacher Gender
Gender	−1.156	−5.972	3.201	−0.774
	(1.727)	(13.570)	(2.544)	(1.840)
Age	0.167	1.619 *	0.262	0.102
	(0.122)	(0.957)	(0.199)	(0.145)
Only Child	1.099	−7.216	2.206	2.090
	(3.425)	(28.699)	(6.727)	(5.672)
Parent Education	−2.256	−22.581 *	−2.696	−3.451 *
	(1.706)	(11.408)	(3.379)	(2.041)
Parent Occupation	−0.444	−2.638	1.873 **	−0.380
	(0.687)	(5.270)	(0.890)	(0.930)
LBC	−1.250	−6.029	−1.469	−0.676
	(0.894)	(6.914)	(1.114)	(1.159)
Family Economic Status	0.424	2.340	−0.646	−0.405
	(0.428)	(3.214)	(0.959)	(0.567)

Standard errors are clustered at the class level and reported in parentheses. Significance: * *p* < 0.10, ** *p* < 0.05.

**Table 4:** Estimation results of instrumental variables.

	^1^ ERM (Eoprobit)		
	FF ^2^	SF ^3^	SD/SFD ^4^
Peer preference for FF	1.192 ***		
	(0.273)		
Peer preference for SF		1.201 *	
		(0.624)	
Peer preference for SD/SFD			1.021 ***
			(0.344)
Control variables	Yes	Yes	Yes
School fixed effects	Yes	Yes	Yes
Observations	1817	1817	1817
	2 SLS		
	FF	SF	SD/SFD
Peer preference for FF	1.137 ***		
	(0.275)		
Peer preference for SF		1.073 *	
		(0.595)	
Peer preference for SD/SFD			1.032 ***
			(0.361)
F test of excluded instruments	222.895	45.398	152.633
Coefficient of instrumental variables in the first stage	−0.454 ***	−0.199 ***	−0.357 ***
	(0.030)	(0.030)	(0.029)
Control variables	Yes	Yes	Yes
School fixed effects	Yes	Yes	Yes
Observations	1817	1817	1817

^1^ ERM refers to the Extended Regression Models. ^2^ FF refers to fast foods, ^3^ SF refers to snack foods, **^4^** SD/SFD refers to soft drinks and sugary fruit drinks. Robust standard errors are reported in parentheses. Significance: * *p* < 0.10, *** *p* < 0.01.

**Table 5:** Mechanism: peer relationships and food availability.

	FF ^1^	SF ^2^	SD/SFD ^3^
Peer preference × peer quantity	0.329 **	0.319 *	0.233
	(0.150)	(0.186)	(0.173)
Control variables	Yes	Yes	Yes
School fixed effects Observations	Yes	Yes	Yes
	1817	1817	1817
Chi ^2^	178.39 ***	108.31 ***	174.57 ***
	FF	SF	SD/SFD
Peer preference × peer quality	0.249 *	0.342 *	0.189
	(0.151)	(0.187)	(0.167)
Control variables	Yes	Yes	Yes
School fixed effects	Yes	Yes	Yes
Observations	1817	1817	1817
Chi ^2^	174.32 ***	100.77 ***	174.22 ***
	FF	SF	SD/SFD
Peer preference × food availability	0.862 ***	0.203	1.255 ***
	(0.245)	(0.273)	(0.294)
Control variables	Yes	Yes	Yes
School fixed effects	Yes	Yes	Yes
Observations	1817	1817	1817
Chi ^2^	194.60 ***	101.38 ***	211.56 ***

^1^ FF refers to fast foods, ^2^ SF refers to snack foods, ^3^ SD/SFD refers to soft drinks and sugary fruit drinks. Robust standard errors are reported in parentheses. Significance: * *p* < 0.10, ** *p* < 0.05, *** *p* < 0.01.

**Table 6:** Heterogeneous effects: individual characteristics.

	FF ^1^	SF ^2^	SD/SFD ^3^
Peer preference × gender (1 = boy)	0.079	−0.315 *	−0.343 **
	(0.145)	(0.180)	(0.162)
Control variables	Yes	Yes	Yes
School fixed effects Observations	Yes	Yes	Yes
	1817	1817	1817
Chi ^2^	168.52 ***	102.36 ***	182.66 ***
	FF	SF	SD/SFD
Peer preference × grade (1 = junior high school)	0.108	0.070	0.498 *
	(0.265)	(0.301)	(0.270)
Control variables	Yes	Yes	Yes
School fixed effects	Yes	Yes	Yes
Observations	1817	1817	1817
Chi ^2^	169.55 ***	95.12 ***	174.58 ***
	FF	SF	SD/SFD
Peer preference × obesity (1 = obese)	0.532 *	−0.098	−0.154
	(0.288)	(0.383)	(0.314)
Control variables	Yes	Yes	Yes
School fixed effects	Yes	Yes	Yes
Observations	1817	1817	1817
Chi ^2^	172.89 ***	100.11 ***	172.22 ***

^1^ FF refers to fast foods, ^2^ SF refers to snack foods, ^3^ SD/SFD refers to soft drinks and sugary fruit drinks. Robust standard errors are reported in parentheses. Significance: * *p* < 0.10, ** *p* < 0.05, *** *p* < 0.01.

**Table 7:** Heterogeneous effects: dietary knowledge for parents.

	FF ^2^	SF ^3^	SD/SFD ^4^
Peer preference × parental DK ^1^	−0.008	−0.052 **	0.015
	(0.020)	(0.026)	(0.025)
Peer preference	1.379 ***	2.423 ***	0.680
	(0.534)	(0.847)	(0.653)
Parental DK	0.020	0.184 **	−0.055
	(0.070)	(0.092)	(0.081)
Control variables	Yes	Yes	Yes
School fixed effects Observations	Yes	Yes	Yes
	1817	1817	1817
Chi ^2^	170.57 ***	99.24 ***	175.91 ***

^1^ DK refers to dietary knowledge. ^2^ FF refers to fast foods, ^3^ SF refers to snack foods, ^4^ SD/SFD refers to soft drinks and sugared fruit drinks. Robust standard errors are reported in parentheses. Significance: ** *p* < 0.05, *** *p* < 0.01.

**Table 8:** Estimation results of different educational styles.

	FF ^1^	SF ^2^	SD/SFD ^3^
Classroom education	−0.009	−0.016	−0.060
	(0.063)	(0.062)	(0.062)
Promotional video or brochure education	−0.057	−0.107 *	−0.121 *
	(0.059)	(0.062)	(0.062)
Practical activity education	0.289 ***	0.373 ***	0.035
	(0.097)	(0.105)	(0.086)
Control variables	Yes	Yes	Yes
School fixed effects Observations	Yes	Yes	Yes
	1817	1817	1817

^1^ FF refers to fast foods, ^2^ SF refers to snack foods, ^3^ SD/SFD refers to soft drinks and sugary fruit drinks. Robust standard errors are reported in parentheses. Significance: * *p* < 0.10, *** *p* < 0.01.

## Data Availability

Data is unavailable due to privacy or ethical restrictions. Readers can contact the author for access to the materials, data, and scripts used in this article.

## Appendix A

**Table A1:** Diet knowledge questionnaire.

NO.	Question	True/False
Q1	Choosing a diet with a lot of fresh fruits and vegetables is good for one’s health.	T
Q2	Eating a lot of sugar is good for one’s health.	F
Q3	Eating a variety of foods is good for one’s health	T
Q4	Choosing a diet high in fat is good for one’s health.	F
Q5	Choosing a diet with a lot of staple foods rice and rice products and wheat and wheat products is not good for one’s health.	T
Q6	Consuming a lot of animal products daily (fish, poultry, eggs and lean meat) is good for one’s health.	F
Q7	Reducing the amount of fatty meat and animal fat in the diet is good for one’s health.	T
Q8	Consuming milk and dairy products are good for one’s health.	T
Q9	Consuming beans and bean products are good for one’s health.	T
Q10	Physical activities are good for one’s health.	T
Q11	Sweaty sports or other intense physical activities are not good for one’s health.	F
Q12	The heavier one’s body is, the healthier he or she is.	F
Q13	Eating salty foods can cause hypertension.	T
Q14	Refined grains (rice and wheat flour) contain more vitamins and materials than unrefined grains.	F
Q15	Lard is healthier than vegetable oils.	F
Q16	Vegetables contain more starch than staple foods (rice or wheat flour).	F
Q17	Eggs and milk are the important sources of high-quality protein.	T
